# The comparison of pregnancy outcomes in fresh and frozen embryo transfer: A cross-sectional study

**DOI:** 10.18502/ijrm.v21i7.13891

**Published:** 2023-08-23

**Authors:** Ramesh Baradaran Bagheri, Mahshid Bazrafkan, Abbas Sabour, Mina Ataei, Bita Badehnoosh, Banafsheh Mashak, Bahareh Khakifirooz, Ramin Moghaddam

**Affiliations:** ^1^Department of Obstetrics and Gynecology, Alborz University of Medical Sciences, Karaj, Iran.; ^2^Reproductive Biotechnology Research Center, Avicenna Research Institute, ACECR, Tehran, Iran.; ^3^Alborz University of Medical Sciences, Karaj, Iran.; ^4^Department of Obstetrics and Gynecology, Social Determinants of Health Research Center, Alborz University of Medical Sciences, Karaj, Iran.; ^5^Department of Obstetrics and Gynecology, Dietary Supplements and Probiotics Research Center, Alborz University of Medical Sciences, Karaj, Iran.; ^6^School of Medicine, Alborz University of Medical Sciences, Karaj, Iran.; ^7^Department of Obstetrics and Gynecology, School of Medicine, Kamali Hospital, Alborz University of Medical Sciences, Karaj, Iran.; ^8^International Academy of Health Sciences Informatics (IAHSI), Geneva, Switzerland.

**Keywords:** Assisted reproductive techniques, In vitro fertilization, Embryo transfer, Cryopreservation, Outcome assessment.

## Abstract

**Background:**

The benefits of frozen embryo transfer (FET) vs. fresh embryo transfer for in vitro fertilization (IVF) have been discussed in previous studies.

**Objective:**

To determine and compare the pregnancy outcomes following FET and frozen embryo transfer in women who underwent assisted reproductive techniques.

**Materials and Methods:**

In this cross-sectional study, 233 women candidates for IVF/intra cytoplasmic sperm injection who referred to the Kamali Training Medical Center, Karaj, Iran during 2019-2020 were evaluated in 2 groups of fresh (n = 127) and frozen (n = 106) embryo transfers. The rates of pregnancy outcomes including chemical and clinical pregnancy, live birth, preeclampsia, ectopic pregnancy, still birth, and pregnancy loss were compared between groups in 3 age subgroups ( < 25, 25-35, and 35-40 yr old).

**Results:**

No significant difference in terms of chemical and clinical pregnancy and live birth rates were observed between groups in women aged < 25 yr. Chemical and clinical pregnancy and live birth rates were significantly higher in the FET group compared to fresh group in 25-35-yr-old women (p = 0.01, p = 0.03, and p = 0.01, respectively). In 35-40-yr-old women, no significant differences were observed in terms of chemical and clinical pregnancy rates, but live birth rate was found to be significantly higher in the FET group (p = 0.02). The pregnancy loss was lower in the FET group (p = 0.038).

**Conclusion:**

In conclusion, the FET method in women aged 25-35 yr significantly increases the chance of successful IVF/intra cytoplasmic sperm injection.

## 1. Introduction

Studies have compared the benefits of frozen embryo transfer (FET) to fresh embryo transfer, including cost-effectiveness (1, 2) and maternal complications during in vitro fertilization (IVF) (3). Studies comparing the outcome of spontaneous vs. assisted reproductive technologies (ART) pregnancies report heterogeneous results. Despite the success of ART to overcome infertility, there is a growing concern regarding both its safety and effect on maternal and child health (4). The most common pregnancy outcomes mentioned in previous studies were ongoing, clinical pregnancy, and abortion rates. A significant rate of ongoing and clinical pregnancy has been reported in FET compared to fresh embryos (5). Another study showed no significant difference in perinatal outcomes between fresh and frozen embryo transfer; however, the live birth rate was slightly increased in fresh cycles, and prematurity was significantly increased among singleton infants in the FET group (6). A systematic meta-analysis and review study showed that the rates of ongoing pregnancy, clinical pregnancy, and abortions are higher with fresh embryos compared with frozen embryos (7). In a double-blind clinical trial, the researchers evaluated 2157 women. The results showed that live-birth rate did not differ significantly between the frozen-embryo group and the fresh-embryo group (48.7% and 50.2%, respectively), but FET resulted in a lower risk of ovarian hyperstimulation syndrome. Also, no significant differences were observed between the 2 groups in the implantation, clinical pregnancy, pregnancy loss, and ongoing pregnancy rates (8).

We need more research on the causes of infertility and treatment methods to improve the success of infertility treatment. This study aimed to determine and compare the pregnancy outcomes following the transfer of fresh or frozen embryos in women in ART cycles during 2019-2020 in Kamali Training Medical Center, Karaj, Iran.

## 2. Materials and Methods

This cross-sectional used the convenience sampling method. 233 women who referred for embryo transfer to Kamali Training Medical Center, Karaj, Iran from April 2019 to April 2020 were divided into 2 groups according to the embryo transfer method until the end of pregnancy. Women < 40 yr with any cause of infertility were included in the study. Women with a history of miscarriage, preterm labor, uterus abnormalities, or underlying medical conditions (e.g., chronic hypertension, diabetes, and lupus erythematosus) and those who refused to share information on pregnancy complications and outcomes were excluded from this study (n = 7).

All participants underwent routine protocol according to their embryo transfer method (fresh/frozen). In this center, embryos were frozen at the cleavage stage using Kitazato Vitrification media (Kitazato, Japan). On the second day of the menstrual cycle, estradiol was given and transvaginal ultrasound was performed after 7-10 days. If the endometrial thickness was > 7 mm, progesterone was administered, and the embryo transfer was performed 3-5 days later.

In the fresh embryo transfer group, transvaginal ultrasound was performed from the 6 ${}^{\text{th}}$ day after gonadotropin administration to assess the size and number of follicles. Once the follicle's size reached > 17 mm, an intramuscular human chorionic gonadotropin (HCG) trigger shot was administered. After 36 hr, using the transvaginal ultrasound-guided follicle puncture method, the oocytes were extracted and incubated with paternal sperms. 3-5 days later, the embryos were transferred into the uterus. In all participants, 14 days after embryo transfer, serum beta human chorionic gonadotropin levels were measured. beta human chorionic gonadotropin ≥ 40 mIU/mL is considered a positive chemical pregnancy. The existence of the fetal heart rate findings in transvaginal ultrasound 4 wk after embryo transfer was considered as a positive clinical pregnancy.

Basic characteristics, including maternal age, body mass index, type of infertility, chemical and clinical pregnancy, first, second, and third-trimester screening results, pregnancy complications (e.g., ectopic pregnancy or preeclampsia, preterm labor, and abortion), and pregnancy outcomes were compared between groups. The missing information about pregnancy outcomes was collected through phone calls.

### Ethical considerations

The protocol of this study was approved by the Ethics Committee of Alborz University of Medical Sciences, Karaj, Iran (Code: IR.ABZUMS.REC.1399.291).

### Statistical analysis

All data were analyzed using IBM-SPSS Statistics 22.0 (IBM, SPCC Inc, USA) with Chi-square test (e.g., demography- for categorical variables), the student *t* test (to compare the means between groups) and the Kolmogorov Smirnov test (K-S test to examine if variables are normally distributed). In all statistical analyses, the significance level of 0.05 was considered.

## 3. Result

A total of 240 women candidates for embryo transfer were included in the study. Finally, 233 women in 2 groups of fresh (n = 127) and FET (n = 106) were studied (Table I).

All quantitative variables had normal distribution (p = 0.05). No significant differences were observed between groups in terms of the mean (standard deviation) age of mothers and fathers, duration of primary infertility, duration of secondary infertility, body mass index, and hormonal profiles (follicle-stimulating hormone, luteinizing hormone, anti-Mullerian hormone) levels. 2 groups showed a significant difference in terms of the number of oocytes and transferred embryos (p < 0.001, and 0.008, respectively) (Table II).

The chemical and clinical pregnancy and live birth rates in the FET group were significantly higher than the fresh embryo group (p = 0.010, 0.022, and 0.003, respectively). No significant differences were observed between groups in terms of pregnancy complications including ectopic pregnancy, preeclampsia, and stillbirth. The pregnancy loss was significantly higher in the fresh group (p = 0.038, Table III).

We divided each group into 3 age subgroups ( < 25 yr, 25-35 yr, and 35-40 yr). No significant difference in terms of chemical and clinical pregnancy and live birth rates were observed between groups in women aged < 25 yr. Chemical and clinical pregnancy and live birth rates were significantly higher in the FET group compared to fresh group in 25-35-yr-old women (p = 0.01, p = 0.03, and p = 0.01, respectively). In 35-40-yr old women, no significant difference was observed in terms of chemical and clinical pregnancy rates, but live birth rate was found to be significantly higher in the FET group (p = 0.02).

**Table 1:** Basic characteristics of participants in 2 study groups


**Variables**		**Fresh**	**FET**	**P-value**
**Age (yr)**				
	**Mother**	32.51 ± 5.8	33.85 ± 5.0	0.06
	**Father**	36.33 ± 5.8	37.69 ± 5.1	0.06
**Infertility type (yr)**				
	**Primary**	5.17 ± 5	4.12 ± 3.8	0.07
	**Secondary**	1.91 ± 0.93	2.12 ± 0.88	0.08
**BMI (kg/m^2^)**		25.31 ± 4.06	26.12 ± 3.01	0.09
**Number of oocytes**		10.58 ± 5.4	8.12 ± 5.7	< 0.001
**Number of transferred embryos**		2.70 ± 0.82	2.42 ± 0.77	0.008
**FSH levels (mIU/mL)**		6.42 ± 2.32	6.98 ± 2.7	0.09
**LH levels (IU/mL)**		5.13 ± 3.60	5.99 ± 3.60	0.07
**AMH levels (ng/mL)**		2.89 ± 2.99	2.23 ± 2.7	0.08
Data presented as Mean ± SD. *t* test. Kolmogorov-Smirnov test was used for normality distribution. BMI: Body mass index, FSH: Follicle-stimulating hormone, LH: Luteinizing hormone, AMH: Anti-Mullerian hormone, FET: Frozen embryo transfer				

**Table 2:** Comparison of pregnancy outcomes between groups


**Outcomes**	**Fresh group (n = 127)**	**FET group (n = 106)**	**P-value**
**Chemical pregnancy**	69 (54.33)	75 (70.75)	0.01 ${}^{*}$
**Clinical pregnancy**	54 (45.21)	61 (57.54)	0.02 ${}^{*}$
**Ectopic pregnancy**	4 (3.14)	3 (2.83)	0.88 ${}^{*}$
**Preeclampsia**	6 (4.72)	4 (3.77)	0.72 ${}^{*}$
**Live birth**	34 (26.77)	48 (45.28)	0.003 ${}^{*}$
**Pregnancy loss**	18 (14.17)	11 (10.37)	0.038 ${}^{*}$
**Still birth**	2 (1.57)	2 (1.88)	0.85 ${}^{†}$
Data presented as n (%). *Chi-square test. † Fisher's exact test. FET: Frozen embryo transfer			

**Table 3:** Comparison of pregnancy outcomes between groups


**Outcomes**		**Fresh group**	**FET group**	**P-value**
**Chemical pregnancy**				
	**< 25 yr**	7 (70)	6 (75)	0.81 ${}^{*}$
	**25-35 yr**	38 (55.88)	35 (79.54)	0.01 ${}^{*}$
	**35-40 yr**	24 (48.97)	34 (62.96)	0.15 ${}^{*}$
**Clinical pregnancy**				
	**< 25 yr**	7 (70)	5 (62.5)	0.73 ${}^{*}$
	**25-35 yr**	26 (38.23)	26 (59.09)	0.03 ${}^{*}$
	**35-40 yr**	21 (42.85)	30 (55.55)	0.19 ${}^{*}$
**Live birth**				
	**< 25 yr**	5 (50)	4 (50)	1.00 †
	**25-35 yr**	19 (27.94)	22 (50)	0.01 ${}^{*}$
	**35-40 yr**	10 (20.40)	22 (40.74)	0.02 ${}^{*}$
Data presented as n (%). *Chi-square test. † Fisher's exact test, FET: Frozen embryo transfer				

## 4. Discussion

The findings of this study showed that the chemical and clinical pregnancy rates in women in the age range of 25-35 yr, who got frozen embryos for IVF were significantly better than in women who got fresh embryos. The live birth rate was significantly better in FET group in the age range of 35-40 yr. The rate of pregnancy loss was higher in the fresh group. No significant differences were observed between the 2 groups (fresh/FET) regarding ectopic pregnancy, preeclampsia, and stillbirth.

According to previous studies, a successful pregnancy depends on coordinating complex biological processes, including interactions between the endometrium and the fetus. In the freezing method, embryos are transferred to the physiological environment of the uterus, while in the fresh method, the endometrium is affected by ovarian hyperstimulation, which causes miscoordination between endometrial receptivity and fetal growth (9, 10). In other words, an inappropriate uterus environment and undesirable endometrial conditions resulting from insufficient endocrine glands secretions lead to abnormal pregnancy outcomes.

Regardless of endometrial receptivity, gene expression is changed in this tissue following hyperstimulation. Another study has shown the effects of these changes in both human and animal endometrial samples (11).

A meta-analysis conducted on 31 studies showed that FET improves pregnancy outcomes, including lower relative risks of placenta previa, placental abruption, low birth weight, very low birth weight, very preterm birth, and perinatal mortality. However, pregnancy-induced hypertension, postpartum hemorrhage, increased in FET group compared with fresh embryo transfer. The risks of gestational diabetes mellitus, preterm premature rupture of the membranes, and preterm birth showed no differences between the 2 groups (12). A systematic review study showed that using the frozen method increased the ongoing pregnancy rate by 32% (95% CI, 1.10-1.59) (6), which is identical to the results of our study. Our study showed better results in the frozen method regarding clinical pregnancy. A similar study showed that the clinical pregnancy rate increased by 31% using the FET method (95% CI, 1.10-1.56) (7). A randomized controlled trial showed that the clinical pregnancy rate increased by 40% through the FET method; therefore, they concluded that FET is the preferred method for IVF/intracytoplasmic sperm injection (5). A review study did not explore a significant difference between the 2 methods of fresh and FET regarding the risk of preeclampsia (13).

The present study was an observational study and the sample size obtained during the study period was insufficient to investigate the pregnancy complications thoroughly, such as preeclampsia, ectopic pregnancy, and stillbirth. More studies with sufficient sample size and in the form of trials are suggested for more definitive conclusions.

## 5. Conclusion

Our study showed that using the FET method significantly increases the chance of successful IVF/ICSI. In other words, using this method increases the rates of chemical and clinical pregnancies and live birth, and reduces the risk of pregnancy loss. We could not recognize the significant difference between the 2 groups regarding ectopic pregnancy, preeclampsia, and stillbirth. Due to a significant reduction in the risk of ovarian hyperstimulation syndrome and improvements in pregnancy outcomes, this study recommends that infertility treatment centers should prioritize the use of frozen methods in their plans. This study also recommends other researchers to conduct similar studies with larger sample sizes to assess the rare outcomes of pregnancy.

##  Conflict of Interest

The authors declared that there was no conflict of interest.
